# Evaluating the Risk of Breast Cancer Recurrence and Metastasis After Adjuvant Tamoxifen Therapy by Integrating Polymorphisms in Cytochrome P450 Genes and Clinicopathological Characteristics

**DOI:** 10.3389/fonc.2021.738222

**Published:** 2021-11-19

**Authors:** Hui Pang, Guoqiang Zhang, Na Yan, Jidong Lang, Yuebin Liang, Xinyuan Xu, Yaowen Cui, Xueya Wu, Xianjun Li, Ming Shan, Xiaoqin Wang, Xiangzhi Meng, Jiaxiang Liu, Geng Tian, Li Cai, Dawei Yuan, Xin Wang

**Affiliations:** ^1^ Department of Medical Oncology, Harbin Medical University Cancer Hospital, Harbin, China; ^2^ Department of Science, Geneis (Beijing) Co., Ltd., Beijing, China; ^3^ Department of Science, Qingdao Geneis Institute of Big Data Mining and Precision Medicine, Qingdao, China; ^4^ Department of Breast Surgical Oncology, National Cancer Center/National Clinical Research Center for Cancer/Cancer Hospital, Chinese Academy of Medical Sciences and Peking Union Medical College, Beijing, China

**Keywords:** breast cancer, hormone receptor-positive, tamoxifen, risk factors, risk-of-recurrence score

## Abstract

Tamoxifen (TAM) is the most commonly used adjuvant endocrine drug for hormone receptor-positive (HR+) breast cancer patients. However, how to accurately evaluate the risk of breast cancer recurrence and metastasis after adjuvant TAM therapy is still a major concern. In recent years, many studies have shown that the clinical outcomes of TAM-treated breast cancer patients are influenced by the activity of some cytochrome P450 (CYP) enzymes that catalyze the formation of active TAM metabolites like endoxifen and 4-hydroxytamoxifen. In this study, we aimed to first develop and validate an algorithm combining polymorphisms in CYP genes and clinicopathological signatures to identify a subpopulation of breast cancer patients who might benefit most from TAM adjuvant therapy and meanwhile evaluate major risk factors related to TAM resistance. Specifically, a total of 256 patients with invasive breast cancer who received adjuvant endocrine therapy were selected. The genotypes at 10 loci from three TAM metabolism-related CYP genes were detected by time-of-flight mass spectrometry and multiplex long PCR. Combining the 10 loci with nine clinicopathological characteristics, we obtained 19 important features whose association with cancer recurrence was assessed by importance score *via* random forests. After that, a logistic regression model was trained to calculate TAM risk-of-recurrence score (TAM RORs), which is adopted to assess a patient’s risk of recurrence after TAM treatment. The sensitivity and specificity of the model in an independent test cohort were 86.67% and 64.56%, respectively. This study showed that breast cancer patients with high TAM RORs were less sensitive to TAM treatment and manifested more invasive characteristics, whereas those with low TAM RORs were highly sensitive to TAM treatment, and their conditions were stable during the follow-up period. There were some risk factors that had a significant effect on the efficacy of TAM. They were tissue classification (tumor Grade < 2 *vs*. Grade ≥ 2, *p* = 2.2e−16), the number of lymph node metastases (Node-Negative *vs*. Node < 4, *p* = 5.3e−07; Node < 4 *vs*. Node ≥ 4, *p* = 0.003; Node-Negative *vs*. Node ≥ 4, *p* = 7.2e−15), and the expression levels of estrogen receptor (ER) and progesterone receptor (PR) (ER < 50% *vs*. ER ≥ 50%, *p* = 1.3e−12; PR < 50% *vs*. PR ≥ 50%, *p* = 2.6e−08). The really remarkable thing is that different genotypes of CYP2D6*10(C188T) show significant differences in prediction function (CYP2D6*10 CC vs. *TT*, *p* < 0.019; CYP2D6*10 CT vs. *TT*, *p* < 0.037). There are more than 50% Chinese who have CYP2D6*10 mutation. So the genotype of CYP2D6*10(C188T) should be tested before TAM therapy.

## Introduction

Hormone receptor-positive (HR+) breast cancer accounts for 75% of all breast cancer patients and is the most common molecular subtype of this disease (1, 2). According to the National Comprehensive Cancer Network 2017 (NCCN2017) (3), HR+ includes estrogen receptor-positive (ER+) and/or progesterone receptor-positive (PR+). Currently, the standard adjuvant endocrine therapy for HR+ breast cancer is 5-year treatment with tamoxifen (TAM) or aromatase inhibitor (AI) (3).

TAM is the earliest and most classical drug in endocrine therapy for breast cancer (4–7). In 1998, The Early Breast Cancer Trialists’ Collaborative Group (EBCTCG) published a meta-analysis of 37,000 randomized clinical trials in 55 groups in *The Lancet*. The study suggested that oral TAM for 5 years in HR+ breast cancer patients can reduce the risk of recurrence of early breast cancer by 47% and the risk of death by 26%, with a survival rate improvement of at least 10 years. The efficacy was independent of age, menstrual status, lymph node metastasis, or prior chemotherapy. In 2011, EBCTCG updated the results, further confirming the efficacy of 5-year TAM treatment after surgery, and a continuing effect until 15 years after surgery. These results established the foundation for oral 5-year TAM as a standard protocol for adjuvant endocrine therapy for breast cancer patients (8). In 2017, EBCTCG studied 88 clinical trials with follow-up over 5 to 20 years, which assessed the risk of breast cancer recurrence in 62,923 patients with at least 5-year TAM treatment. It was found that even in patients with low histological grade of T1N0, 10% of patients had developed distant metastasis 20 years later. Therefore, it is necessary to prolong the time of endocrine therapy or strengthen endocrine therapy for patients with high risk of recurrence (9).

The same dose of TAM (10 mg, b.i.d.) was administrated to patients, however, with significantly different effectiveness in individual patients (10), which presents the need for precision medicine (11, 12). This individualized difference in effectiveness could not be fully explained by liver and kidney function, age, lifestyle or a combination of medication, and patient compliance. Genetic factors might play an important decisive role (13, 14). A number of studies have shown that TAM metabolized through the cytochrome P450 enzymes of the liver to the active products 4-hydroxytamoxifen and endoxifen to play pharmacological effects. However, the cytochrome P450 enzyme activity is influenced by its genetic polymorphism.

CYP2D6 is a key enzyme in the metabolic process of TAM, and the relationship between its genetic polymorphism with TAM metabolism and efficacy has attracted much attention (13, 15). Several studies have shown that the CYP2D6 enzymatic activity in breast cancer patients with CYP2D6*3(775delA), CYP2D6*4(G506-1A), CYP2D6*5 (fragment deletion), CYP2D6*10(C188T), and CYP2D6*41 (c.985+39G>A) alleles is reduced; the levels of activated intermediate metabolites 4-hydroxytamoxifen and endoxifen are decreased after TAM treatment; and the recurrence rate of breast cancer is higher, while the survival rate after recurrence is lower (16–19). Among Chinese population, the distribution frequency of CYP2D6*10 was shown to be as high as 50%, which was thought to be a major factor affecting *in vivo* activation efficiency of TAM (20). The serum concentrations of endoxifen in breast cancer patients with CYP2D6*1/*10(CT) and CYP2D6*10/*10(TT) were shown to be decreased more significantly than those of individuals with wild-type CYP2D6 (10).

CYP2C19 is a typical CYP450 enzyme that affects the metabolism of TAM transforming into 4-hydroxytamoxifen, and it also participates in the metabolism of estradiol and estrone (16–18). The enzymatic activity of CYP2C19 in patients with CYP2C19*2(G681A) and *3(G636A) alleles is decreased, and the 5-year disease-free survival (DFS) rate is lower than that in patients with CYP2C19 wild type (21–23). However, some studies suggest that the activity of CYP2C19 in breast cancer patients with CYP2C19*17(C-806T) allele was enhanced, and the application of TAM treatment was beneficial to those patients (16, 24).

*N*-Demethylation of TAM is mainly mediated by CYP3A5, and CYP3A5*3(A6986G) mutation reduces the enzymatic activity (9, 25). Goetz et al. showed that the DFS time, DFS rate, and overall survival (OS) rate of breast cancer patients with different CYP3A5 genotypes were similar (26). However, another study (27) found that the recurrence risk of individuals with CYP3A5*3/*3(GG) is significantly decreased after 5 years of TAM therapy, suggesting that CYP3A5 polymorphism might also be an important factor affecting the efficacy of TAM.

In summary, there lacks a unified quantitative indicator to predict the superiority of TAM in the treatment of patients with early HR+ breast cancer. There lacks predictive model to specifically differentiate the patients with recurrence risk after early TAM treatment. We aimed to develop such a model to more efficiently guide such patients for improved DFS from individualized TAM therapy.

## Materials and Methods

### Patient Selection

In this retrospective study, patients’ information was retrieved from the sample database of the Galactophore Department, Cancer Hospital Affiliated to Harbin Medical University. The keywords used to screen patients from the sample storage management system included invasive breast cancer, HR+, endocrine therapy, and TAM. Briefly, 5,731 patients were retrieved. Among these, patients were excluded based on the following criteria (**Figure S1**): 1) patients without clinicopathological information or incomplete clinical pathology information; 2) patients with HER2 (3+) or HER2 fluorescence *in situ* hybridization (FISH) (+) who received trastuzumab treatment; 3) patients without disease progression treated with toremifene or AIs; 4) patients without blood samples; or 5) patients with failed repeated extraction of blood sample. Finally, 256 patients were included in the study.

This was a retrospective study. Informed consent from patients was not required in this study. All samples were retrieved from the sample library of Cancer Hospital Affiliated to Harbin Medical University. This study was approved by the Ethics Committee of Cancer Hospital Affiliated to Harbin Medical University (Ethical No. KY2017-03).

This study involved the clinical information and pathological data that might be related to the incidence of recurrence and metastasis, including tissue classification, the maximum diameter of tumor, the number of lymph node metastases, whether the patients were in menopause or not, patient’s age, and the expression levels of ER, PR, HER2, and Ki67. Recurrence and metastasis were defined as the recurrence of primary lesions, metastasis of axillary lymph nodes, mammary glands, and distant organs. All the patients received TAM 10 mg each time, twice daily. In addition, all patients’ information and blood samples used in this study were obtained following the approval from the hospital.

### Detection of Genetic Polymorphism

In recent years, time-of-flight mass spectrometry (TOFMS) has become a very effective method for genotyping single-nucleotide polymorphisms (SNPs). TOFMS can detect genotypes rapidly and efficiently (28). Several SNP genotyping methods have been implemented with a high degree of automation and are being applied for large-scale association studies. It is the working principle of TOFMS. Firstly, a segment of DNA containing SNP site was amplified by PCR (about 50 bp before and after SNP site). And then SAP enzyme was used to remove the dNTP and the primers in the PCR system. A single-base extension primer was added in which three “terminal base” was next to SNP site and used four kinds of ddNTP instead of dNTP (ddNTP corresponds to the allele of SNP locus). So only one base is extended at the SNP locus. TOFMS was used to detect the difference of the molecular weight between the extended product and the non-extended primer, which can determine the base at this point (29, 30).

Genetic polymorphisms were assessed using DNA extraction from retained blood samples and were examined based on TOFMS platform and multiplex long PCR. TOFMS platform obtained the genotypes at nine loci of three genes at a time. We designed three primers for each site, and the primer sequence information was provided in the **Supplementary Materials** (**Table S1, S2**). The primers were designed by an online software named Agena Bioscience (www.agenacx.com). The deletion of CYP2D6*5 fragments was obtained by multiplex long PCR.

### Derivation of the Prediction Model

**Figure 1:**
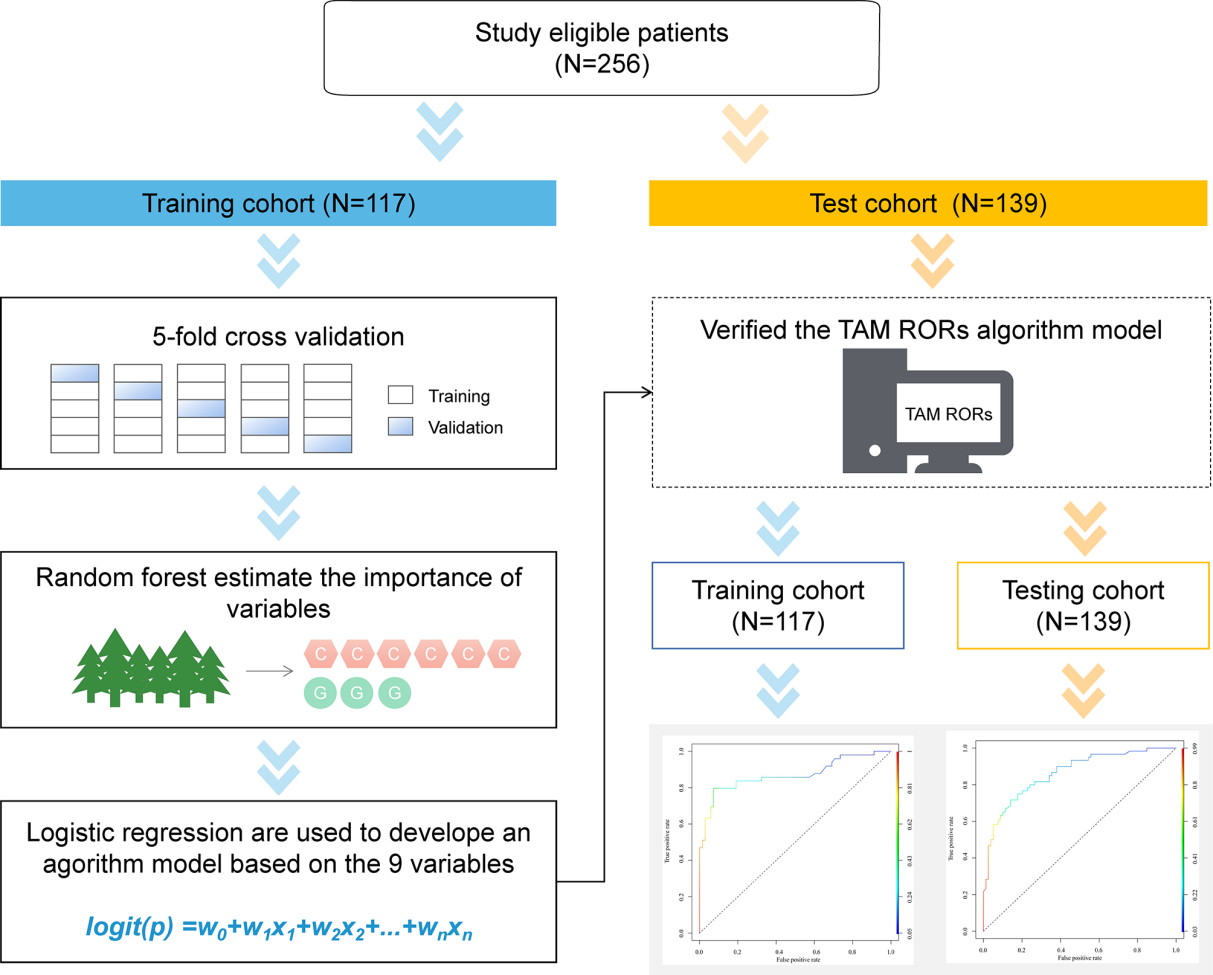
Schematic for development of TAM RORs. In total, 256 patients were eligible for analysis. Samples were split into training and independent test sets by the surgery time. The training set was used to tune the parameters and select the best model using five-fold cross-validation. After training, the test set was used to independently assess the performance of the final model. TAM RORs, tamoxifen risk-of-recurrence score.

A total of 256 patients who met the inclusion criteria were divided into the training cohort and test cohort according to the surgery time before or after June 1, 2013 (**Figure 1** and **Figure S2**). A total of 117 patients were assigned to the training cohort, while the remaining 139 patients were assigned to the test cohort (**Table S6**). The training cohort was used to analyze the correlation between clinicopathological factors, gene locus polymorphism, the recurrence and metastasis of disease. The test cohort was used to test the performance of the algorithm model. The training cohort and test cohort both included patients who developed recurrence and metastasis, as well as those without disease progression during 5 years of clinical follow-up. All patients’ clinical information and detection results of genetic polymorphism were assigned a value based on the degree of disease malignancy and TAM metabolic enzyme activity, which was used for subsequent mathematical statistics (**Table 1** and **Tables S1**, **S3–S5**).

**Table 1:** Patients’ information and tumor characteristics.

Characteristic (N = 256)	No. of patients	Percentage (%)	
Age			Median age, years
<40 years	26	10.16%	48 years (25–76)
≥40 years	230	89.84%	
Menopause			
Premenopause	146	57.03%	
Postmenopause	110	42.97%	
Tumor size			
<2 cm	103	40.23%	
≥2 cm	153	59.77%	
Tumor grade			
I, I~II, II	177	69.14%	
II~III, III	79	30.86%	
Lymph node metastasis			
Node-Negative	112	43.75%	
<4	56	21.88%	
≥4	88	34.38%	
ER status			
<50%	48	18.75%	
≥50%	208	81.25%	
PR status			
<50%	102	39.84%	
≥50%	154	60.16%	
Ki67 status			
≤20%	182	71.09%	
20%~30%	46	17.97%	
≥30%	28	10.94%	
HER2 status (IHC and FISH)			
−; +; 2+ and FISH (−)	229	89.45%	
3+ or FISH (+)	27	10.55%	

ER, estrogen receptor; PR, progesterone receptor; FISH, fluorescence in situ hybridization; IHC, immunohistochemistry.

We constructed a model to predict the risk of breast cancer recurrence and metastasis by clinicopathological factors and gene locus polymorphism. The Random Forest algorithm was used for assessing the importance of all known features. We selected top features according to MeanDecreaseGini score of the Random Forest algorithm. The top features were used as the input for further logistic regression analysis to predict the risk of breast cancer recurrence and metastasis.

There are hyper-parameters in our model including number of features, tree number, and link function. A grid search algorithm was used to select the hyper-parameters as below.

Feature Number = [3, 6, 9, 12, 15, 18]
Tree Number = [1000, 10000, 20000]
Feature Importance Index = [“MeanDecreaseGini”, “MeanDecreaseAccuracy
(absolute value)”, “MeanDecreaseAccuracy”].

We used the five-fold cross-validation to select these hyper-parameters. The results are shown in **Table 2**. As a result, we set the feature number to be 9, Feature Importance Index to be “MeanDecreaseGini,” and Tree num to be 20,000 (**Table 2**). After that, the model with these hyper-parameter set was trained by the whole training dataset. The whole process can be found in **Table 3**.

**Table 2:** Hyper-parameter selection by cross-validation.

Feature importance index	Tree num	Feature number selection					
		3	6	9	12	15	18
MeanDecreaseGini	1,000	0.67	0.79	0.85	0.82	0.81	0.81
	10,000	0.74	0.81	0.87	0.83	0.82	0.81
	20,000	0.74	0.81	0.87	0.83	0.82	0.81
MeanDecreaseAccuracy (absolute value)	1,000	0.67	0.79	0.85	0.81	0.81	0.81
	10,000	0.74	0.81	0.85	0.83	0.82	0.81
	20,000	0.74	0.81	0.86	0.83	0.82	0.81
MeanDecreaseAccuracy	1,000	0.71	0.8	0.83	0.81	0.79	0.77
	10,000	0.74	0.82	0.83	0.81	0.8	0.8
	20,000	0.74	0.82	0.83	0.81	0.8	0.8

**Table 3:** The process of building TAM RORs model.

Algorithm Build TAM RORs model
Input: Training dataset Output: Trained model and performance of the model
**1** for each one hyper-parameter set in hyper-parameters grid do **2** for 5-fold cross validation process do **3** select features in Random forest by the hyper-parameter set; **4** train logistic regression model; **5** compute AUC from ROC curve for one subset of cross validation **6** Add the new result to the result of this validation process **7** sort the performance of hyper-parameter set and store optimal set; **8** select the optimal hyper-parameter data set to be the hyper-parameter of model; **9** train the model with whole training data set; **10** final; **11** return model and AUC value of ROC curve;

TAM RORs, tamoxifen risk-of-recurrence score.

In the training cohort (N = 117), the Random Forest algorithm was used for the importance assessment of 19 variables including patient’s age, whether the patient was menopausal or not, the number of lymph node metastases, the maximum diameter of tumor, tissue classification, and the expression levels of ER, PR, HER2, Ki67, CYP2D6 *2, *3, *4, *5, *10, *41, CYP2C19 *2, *3, *17, and CYP3A5*3 (**Figure S3**). Ten variables with MeanDecreaseGini score less than 2.1 were excluded; the remaining nine variables were used for further logistic regression analysis. As a result, TAM RORs (1) = −2.74 + 3.54Grade + 0.75LN + 0.28CYP2C19*2 + 0.49PR + 0.31CYP2D6*10 + 1.11ER − 0.1CYP3A5*3 − 0.28Ki67 − 0.37Size was obtained. Then, TAM RORs (1) were converted into binary results. Specifically, a patient is considered to have high risk of breast cancer recurrence and metastasis if TAM RORs (2) = 1/(1 + e-TAM RORs(1)) is greater or equal to 0.175 and have low risk if the value is less than 0.175. The cutoff 0.175 is trained by the training dataset, by which we obtained a training area under the curve (AUC) of 0.87. Lastly, the TAM RORs of each patient in the test cohort were calculated to verify the performance of the model in an independent testing data (AUC = 0.86) (**Table S7** and **Figure S4**). We also tested the other two methods: neural network and support vector machine (SVM).

SVM is one of the popular supervised learning algorithms. It is used for Classification as well as Regression problems. Primarily, it is used for Classification situation in machine learning. The aim of the SVM algorithm is to find the best line or decision boundary, which can divide n-dimensional space into classes in order to put the new data point in the correct space easily in the future. This best decision boundary is termed a hyperplane. SVM selects the extreme vectors that help in finding the hyperplane. These extreme cases are named as support vectors, and then the algorithm is termed as SVM.

Neural networks are used almost in every machine learning application because of their reliability and mathematical power. Each neuron in the neural networks is divided into different groups according to the order of receiving information. Each group can be regarded as a neural layer. The neurons in each layer receive the output of the neurons in the previous layer and output to the neurons in the next layer. The information in the whole network propagates in one direction, and there is no reverse information propagation. The feedforward network can be represented by a directed acyclic graph. The feedforward network can be regarded as a function, and the complex mapping from input space to output space is realized through the multiple compositions of simple non-linear functions. The network structure is simple and easy to implement.

In this article, we applied four-level neural networks on our classification problem by using R programming.

R version 3.4.3 (2017-11-30) was used for the classification and training of the prediction model (**Figure 1**).

### Statistical Analysis

The statistical package stats of R version 3.4.3 software was used for statistical analysis. We studied the difference of clinicopathological variables or genotypes between two groups with different TAM RORs value. Specifically, we conducted Wilcoxon’s test, adjusted the *p*-values by the Benjamini–Hochberg method, and added the *p*-values to ggplot for box blots and dot plots (**Figures S6**, **S7**). Friedman’s test was adopted to discover the significant difference between logistic regression, the SVM algorithms and Feedforward Neural Network and the compared algorithms on the test dataset.

## Results

### A Logistic Regression Model to Predict the Performance of Tamoxifen Adjuvant Therapy

In the training cohort (N = 117), 75 (64.10%) patients obtained high TAM RORs (2) scores, while 42 (35.90%) patients obtained low TAM RORs (2) scores. Among 75 patients with high TAM RORs (2) scores, 42 (56.00%) patients developed recurrence and metastasis after TAM treatment. Among 42 patients with low TAM RORs (2) scores, 35 (83.33%) patients had no disease progression during the follow-up period. Therefore, we speculated that patients with high TAM RORs (2) scores had poor prognosis and were more likely to exhibit invasive tumor characteristics, while those with low TAM RORs (2) scores had good prognosis and stable disease control after TAM treatment (**Table 4**).

**Table 4:** TAM RORs predicts recurrence and metastasis in breast cancer.

	High TAM RORs	Low TAM RORs
Training cohort (N = 117)	75 (64.10%, 75/117)	42 (35.90%, 42/117)
Recurrence and metastasis	42 (56.00%, 42/75)	7 (16.67%, 7/42)
Disease progression-free	33 (44.00%, 33/75)	35 (83.33%, 35/42)
Test cohort (N = 139)	80 (57.55%, 80/139)	59 (42.45%, 59/139)
Recurrence and metastasis	52 (65.00%, 52/80)	8 (13.56%, 8/59)
Disease progression-free	28 (35.00%, 28/80)	51 (86.44%, 51/59)
Sensitivity	86.67% (52/52 + 8)	
Specificity	64.56% (51/51 + 28)	

TAM RORs, tamoxifen risk-of-recurrence score.

**Figure 2:**
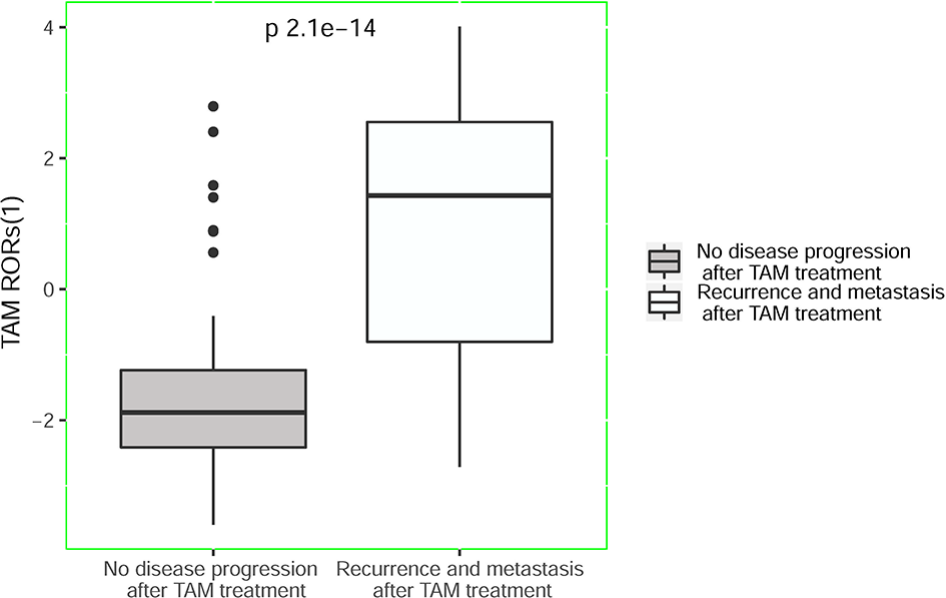
TAM RORs predicts recurrence and metastasis in breast cancer. Box-whisker plots of TAM RORs (1) values. TAM RORs, tamoxifen risk-of-recurrence score.

**Figure 3:**
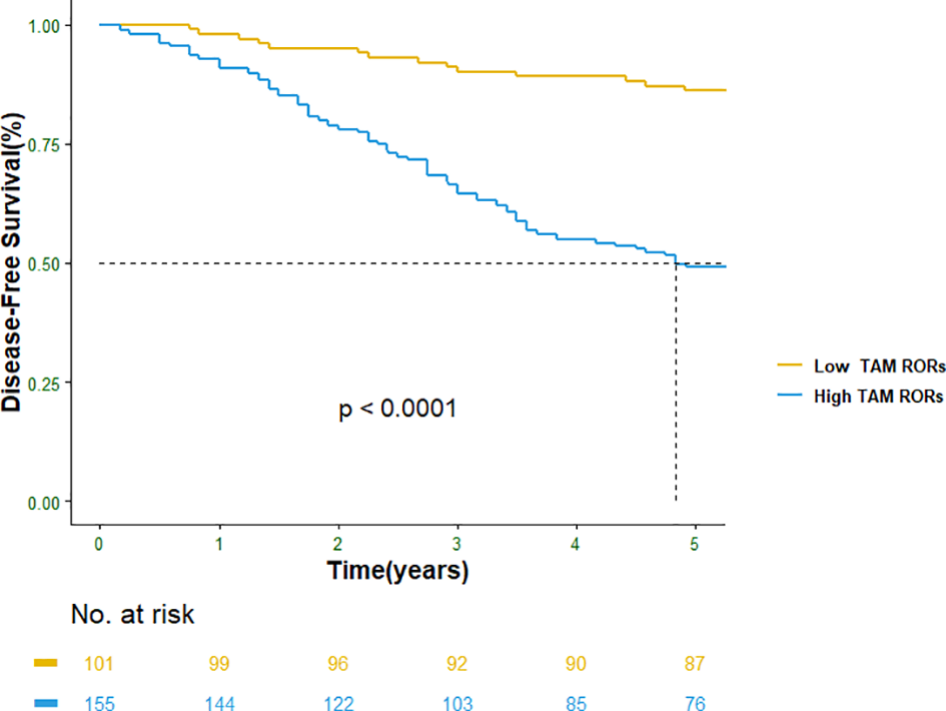
Disease-free survival (DFS) in TAM RORs. TAM RORs, tamoxifen risk-of-recurrence score.

In the test cohort, the recurrence and metastasis probability of breast cancer patients after TAM treatment was evaluated, and our hypothesis was verified. Among 139 patients in the test dataset, 80 (57.55%) patients obtained high TAM RORs, while 59 (42.45%) patients obtained low TAM RORs. Among 80 patients with high TAM RORs, 52 (65.00%) developed recurrence and metastasis after TAM treatment. In addition, among 59 patients with low TAM RORs, 51 (86.44%) patients did not have disease progression during the follow-up period. Therefore, we verified that the sensitivity and specificity of TAM RORs (2) were 86.67% and 64.56%, respectively (**Table 2**). Moreover, our hypothesis was verified: breast cancer patients with high TAM RORs (2) were always less sensitive to TAM treatment and had tumor invasion occurrence. Conversely, breast cancer patients with low TAM RORs have always high sensitivity to TAM treatment and had stable disease control (**Figures 2** and **3**, **Table S4**, **Figure S5**).

### Major Risk Factors for Tamoxifen Resistance

According to the TAM RORs value, the effects of breast cancer patients’ information and tumor characteristics on the efficacy of TAM were observed. Among patients’ information and their tumor characteristics, four factors including the number of lymph node involvement, the expression levels of ER and PR, tumor diameter, and tumor tissue classification had a significant effect on the efficacy of TAM (Node-Negative *vs*. Node < 4, *p* = 5.3e−07; Node < 4 *vs*. Node ≥ 4, *p* = 0.003; Node-Negative *vs*. Node ≥ 4, *p* = 7.2e−15; ER < 50% *vs*. ER ≥ 50%, *p* = 1.3e−12; PR < 50% *vs*. PR ≥ 50%, *p* = 2.6e−08; tumor Size < 2cm *vs*. tumor Size ≥ 2cm, *p* = 0.013; tumor Grade < 2 *vs*. Grade ≥ 2, *p* = 2.2e−16; **Figure S6**). Similarly, the effect of TAM metabolism-related gene locus polymorphisms on the efficacy of TAM in breast cancer patients was observed. We detected 10 loci in CYP2D6, CYP3A5, and CYP2C19. Among these loci, three loci, i.e., CYP2D6*10(C188T), CYP2C19*2(G681A), and CYP3A5*3(A6986G), had a significant effect on the efficacy of TAM (CYP2D6*10(C188T): *CC vs*. *TT*, *p* < 0.019; *CT vs*. *TT*, *p* < 0.037; CYP2C19*2(G681A): *GG vs*. *GA*, *p* < 0.02; *GG vs*. *AA*, *p* < 0.0043; CYP3A5*3(A6986G): *AG vs*. *GG*, *p* < 0.0029; *AA vs*. *GG*, *p* < 0.038; **Figure S7**). There were significant differences between wild type and CYP2D6*10/*10 (*p* < 0.019), as well as between CYP2D6*1/*10 and CYP2D6*10/*10 (*p* < 0.037). Patients with CYP2D6*10 were more likely to obtain higher TAM RORs (1) scores, and their disease progression occurred during the follow-up period. There are more than 50% Chinese whose have CYP2D6*10 mutation. So it is very important to know which genotypes CYP2D6*10(C188T) patients have. The genetic polymorphism of this locus significantly affected the activation efficiency of TAM *in vivo* and is an important predictor of prognosis in TAM treatment. There was also a significant difference between wild type and CYP3A5*1/*3 (*p* < 0.038). However, a significant difference existed between CYP3A5*1/*3 and CYP3A5*3/*3 (*p* < 0.003): 53.08% CYP3A5*3/*3 patients obtained low TAM RORs, and no disease progression was observed during the follow-up period. The results were similar to those reported by Wegman *et al.*, who suggested that the risk of recurrence in individuals with CYP3A5*3/*3 significantly decreases after 5 years of TAM treatment (27).

### Comparison of the Predictive Effectiveness

Clinically, STEPP analysis is used to predict the risk of recurrence in premenopausal patients with HER2-negative /HR+ early breast cancer. The prediction results of Subpopulation Treatment Effect Pattern Plot (STEPP) analysis were used to determine whether Ovarian Function Suppression (OFS) should be performed in conjunction with endocrine therapy. However, this method only involves clinicopathological indicators, and it did not take into account the impact of TAM metabolism.

We screened 132 patients with HER2-negative premenopausal breast cancer from 256 patients. Among them, 44 patients had breast cancer recurrence, whereas 88 patients had no recurrence or metastasis in 5 years of follow-up. The prognosis was predicted by STEPP score. Among the 44 patients with breast cancer recurrence, 39 patients were diagnosed as “medium-high risk,” and 34 patients as “low risk” among the 88 patients without recurrence and metastasis. The predictive sensitivity was 88.64% and specificity was only 38.64% in the screened 265 patients. The prognosis was predicted by TAM RORs. Among the 44 patients with breast cancer recurrence, 38 patients with high risk of recurrence were identified, and 40 patients with “low risk” of recurrence were identified among 88 patients without recurrence metastasis. The predictive sensitivity of TAM RORs in 265 patients reached 86.36% and specificity reached 54.54%. The comparison of the two formulas can be found to be equally excellent in predicting patients with high recurrence risk. However, in the prediction of low-risk patients, TAM RORs showed better specificity.

We have tested other two methods among which deep learning has the best performance (AUC: 0.88). The sensitivity and specificity of SVM are lower than those of the logistic regression. That is, the predictive ability of SVM is inferior to that of the logistic regression. On the other hand, the performance of Fully Connected Feedforward Neural Network is slightly better than that of the current model.

To judge whether or not our approaches were statistically significant, Friedman’s test was conducted at α = 0.05 in terms of TAM RORs from 139 patients in the test dataset. TAM RORs of each patient from the three algorithms were the data used to conduct Friedman’s test. The results of algorithms on 139 patients of the test dataset are shown in **Table 5**. Here, χ^2^ is the chi-square, *df* is the degree of freedom, and *p* is the *p*-value. Friedman’s test results told us that there was no strong significant difference between the three compared algorithms because *p*-value was not less than the specific value of alpha, which is set to be 0.05.

**Table 5:** Results of Friedman’s test between our approaches and the three compared algorithms.

	Friedman’s test		
	χ^2^	*df*	*p*
TAM RORs	0.23864	2	0.8875

TAM RORs, tamoxifen risk-of-recurrence score.

Friedman’s test results told us that there was no strong significant difference between the three compared algorithms. However, the Fully Connected Feedforward Neural Network is implicit and has explicit formula for predicting, which is difficult in mining the biological mechanisms behind the prediction and it. Thus, we finally chose the logistic regression in this study.

## Discussion

TAM, as a standard drug for the endocrine therapy of breast cancer (3), effectively reduces the recurrence and mortality of HR+ breast cancer patients (4, 8, 31, 32). Nonetheless, 50% of the patients did not benefit from it, which presents a great concern in clinical practice (33–35). The prognosis of ER-positive breast cancer patients has always been a hot topic in clinical research. At present, there are five recognized clinical prediction methods (3, 36–41): 1) Oncotype Dx recurrence score (RS), 2) PAM50-based Prosigna risk of recurrence (ROR), 3) Breast Cancer Index (BCI), 4) EndoPredict (EPclin), and 5) MammaPrint Netherland Kanker Institute 70-gene signature. These prognosis prediction methods are all based on multigene expression profiles. Compared with independent clinical factors, multigene expression profiles combined with clinical factors have shown significantly predictive efficiency (42). Nevertheless, following the progression of TAM metabolic mechanism research, it has been shown that being the activation product of TAM, endoxifen has high activity to inhibit the growth of tumor by competitive binding of ER with estradiol and blocking estrogenic effect. This process is regulated by multiple enzymes including CYP2D6, CYP2C19, and CYP3A5 (43–45). The activities of these enzymes are influenced by the genetic polymorphism at different loci, thus showing individual differences (23–25). These enzymes further reveal the individual differences in drug sensitivity of TAM and the efficacy of treatment. At present, the genetic polymorphisms affecting the metabolic efficiency of TAM had not been covered by any algorithm model of clinical prognostic assessment, which can be interpreted as a knowledge gap to predict the prognosis of ER-positive breast cancer patients with TAM treatment (36–41, 46–49). The present study was based on the factors affecting TAM metabolic efficiency, combined with clinicopathological information necessary to establish an algorithm model to evaluate the benefit of patients after TAM treatment.

The efficacy of TAM was predicted by three methods, which have the same training set and test set. They are Logistic Regression, SVM, and Fully Connected Feedforward Neural Network. In SVM, when kernel = “linear,” the sensitivity and specificity are 58% and 92%; when kernel = “sigmoid,” the sensitivity and specificity are 58% and 92%; and when kernel = “radial,” the sensitivity and specificity are 65% and 89.9%. The predictive ability of SVM is inferior to that of the logistic regression. In the Fully Connected Feedforward Neural Network, set the number of network layers to 4 and the middle-hidden layers to 2, the sensitivity and specificity are 86.67% and 73.41%, respectively. On the other hand, Friedman’s test results told us that there was no strong significant difference between our approaches and the three compared algorithms. Since the Fully Connected Feedforward Neural Network has bad interpretability, in considering the practical application, we finally chose the logistic regression.

There were limitations in this study. This was a retrospective study based on the collection of clinical samples. Although we have included as much as possible of the available clinical information, the clinical treatments for invasive breast cancer still had a certain impact on the study; e.g., after surgical treatment, patients received routine chemotherapy before they underwent TAM endocrine therapy. This might explain why a small number of patients had high TAM RORs, suggesting a higher risk of recurrence, but there was no disease progression found in actual clinical follow-up. Nonetheless, although the datasets had some limitations, they performed well in the current verification. We also tried to enroll as many patients as possible in the study to further verify our TAM RORs. In addition, we adopted a simple logistic regression model to perform the prediction, which might not be optimal. Deep learning and network-based methods have been proven to be effective in many similar prediction problems (50–55), which will be tested in the future to improve the prediction accuracy.

In the analysis of genetic polymorphisms in 256 patients, we found that the genotype frequency of CYP2C19*2(G681A) was different from that in the National Center for Biotechnology Information (NCBI) SNP database. It was CYP2C19*2(G681A) AA genotype (in our study: 7.03% *vs*. China population frequency from NCBI: 0%) (**Table S8**). We collected 243 blood samples from healthy individuals in Northeastern China and verified this phenotype: CYP2C19*2(G681A) AA genotype 8.2%. It shows the distribution characteristics of CYP2C19*2(G681A) in Northeast China.

Over the recent years, with the rapid development of precision medicine, gene detection has become more and more important for cancer diagnosis, prognosis, and drug selection (56–62). Several studies have shown that the metabolic efficiency of TAM was related to the genetic polymorphisms of certain P450 enzymes, thereby affecting the efficacy of drug therapy. Based on this theory, our TAM RORs can be used to predict the efficacy of TAM treatment and improve personalized endocrine therapy in patients with invasive breast cancer.

## Data Availability Statement

The original contributions presented in the study are included in the article/**Supplementary Material**. Further inquiries can be directed to the corresponding authors.

## Ethics Statement

Written informed consent was obtained from the individual(s) for the publication of any potentially identifiable images or data included in this article.

## Author Contributions

Conception and design: LC, DY, and XWa. Study development and methods: NY, GZ, and HP. Medical and technical support: DY, NY, and HP. Collection and assembly of data: GZ, HP, XX, YC, GT, XWu, XL, MS, and XQW. Data analysis and interpretation: NY, JDL, YBL, XM, and JXL. Manuscript writing: NY, XWa, GZ, and HP. All authors contributed to the article and approved the submitted version.

## Funding

This study was funded by the National Key Research and Development Program of China (Grant No. 2019YFE0110000), National Natural Science Foundation of China (Grant No. 82072097), Science and Technology Fund of Heilongjiang Province of China (Grant Number LH2020H126), and Hai Yan Fund from Harbin Medical University Cancer Hospital [Grant Number JJZD2020-06], and National Cancer Center climbing Foundation (NCC201808B014).

## Conflict of Interest

NY, JDL, YBL, XQW, GT, and DY were employed by company Geneis (Beijing) Co. Ltd.

The remaining authors declare that the research was conducted in the absence of any commercial or financial relationships that could be construed as a potential conflict of interest.

## Publisher’s Note

All claims expressed in this article are solely those of the authors and do not necessarily represent those of their affiliated organizations, or those of the publisher, the editors and the reviewers. Any product that may be evaluated in this article, or claim that may be made by its manufacturer, is not guaranteed or endorsed by the publisher.
