# Supplementary material for: Evaluating the Risk of Breast Cancer Recurrence and Metastasis After Adjuvant Tamoxifen Therapy by Integrating Polymorphisms in Cytochrome P450 Genes and Clinicopathological Characteristics
Source: Front Oncol. 2021 Nov 19;11:738222. doi: 10.3389/fonc.2021.738222 (PMC8639703; doi:10.3389/fonc.2021.738222)
Supplement: Supplementary file 1 [file DataSheet_1.pdf]

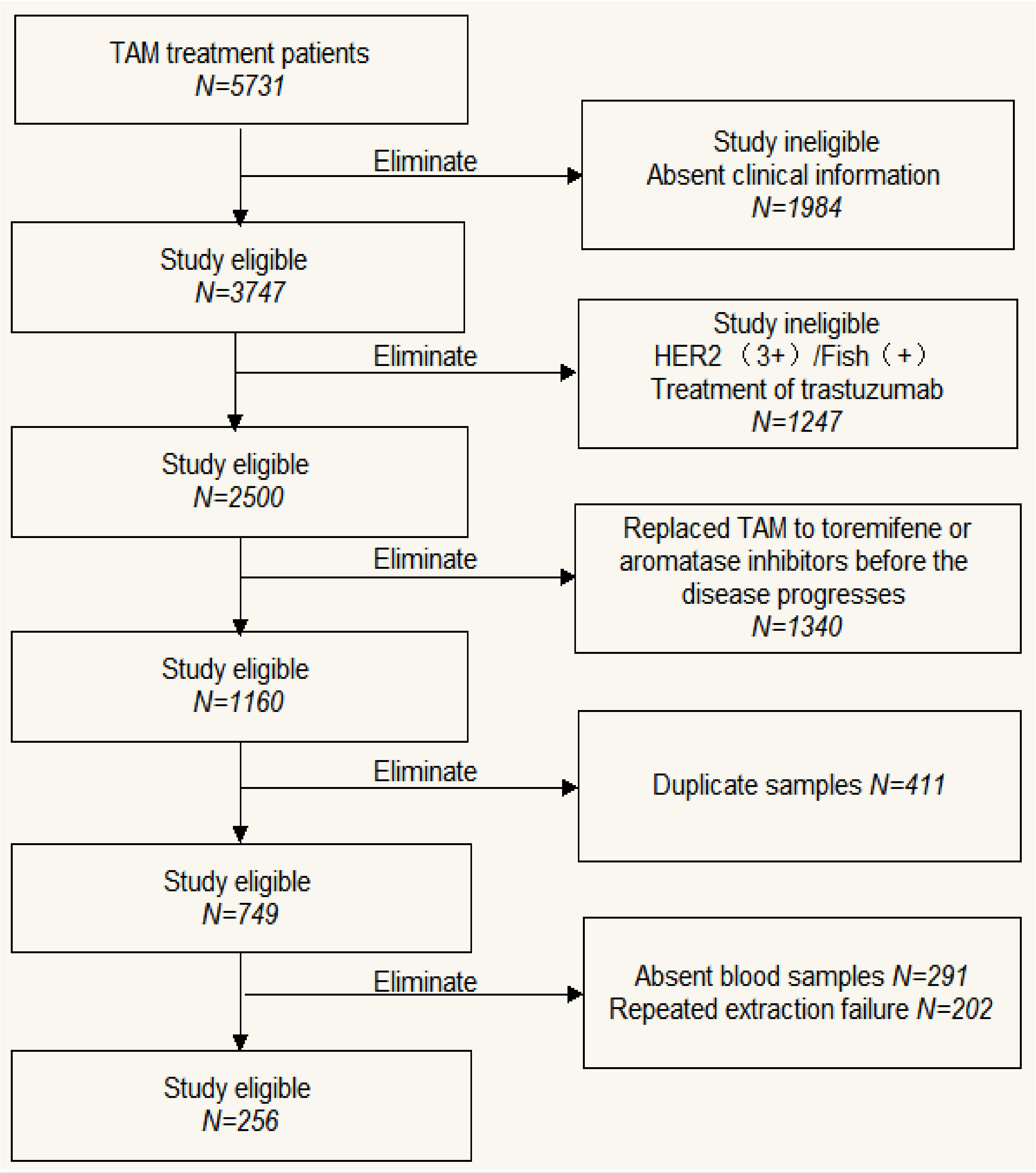

Figure S1. Study flow chart for selection of breast cancer patients. Patients were eligible for study based on specific exclusion criteria.

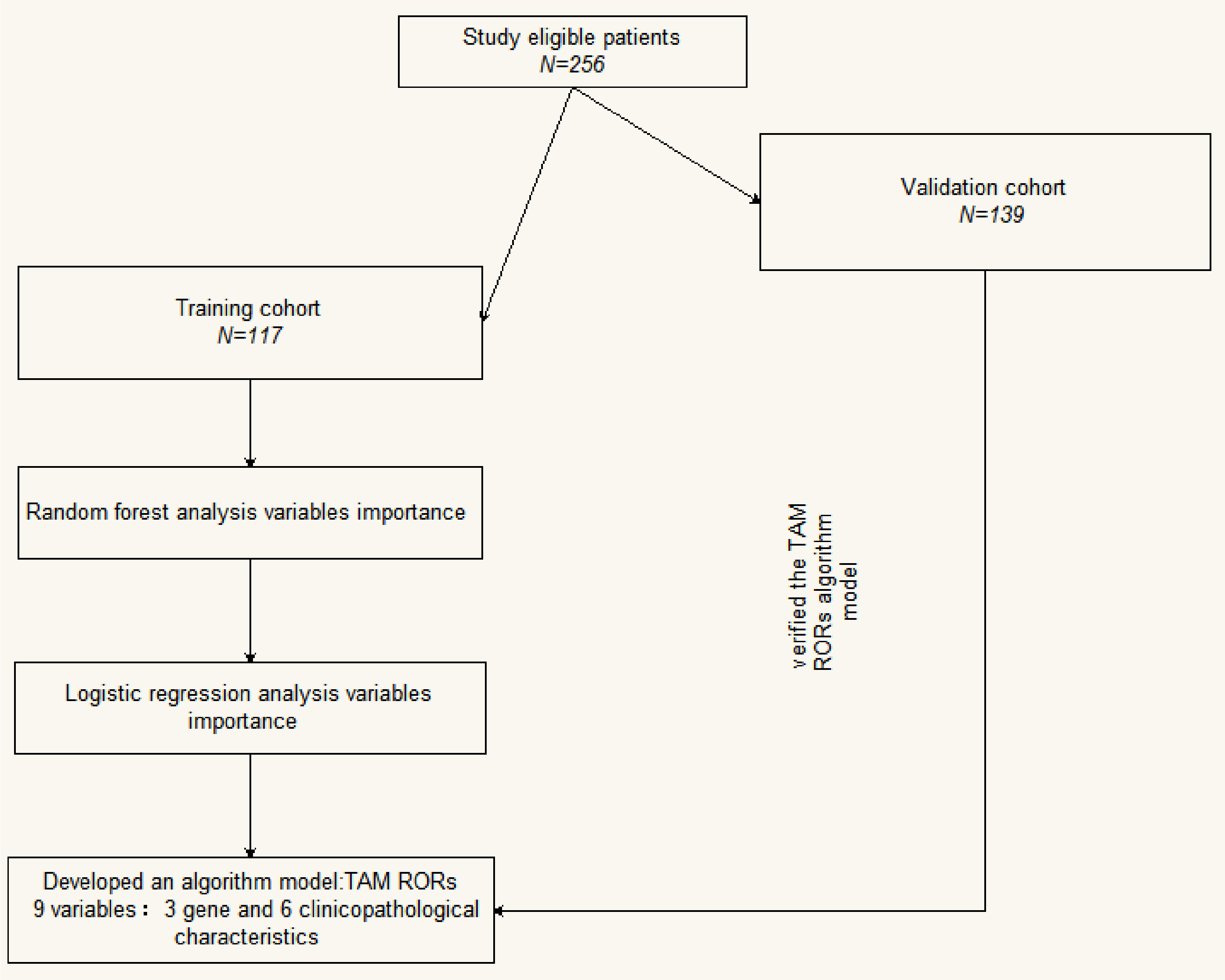

Figure S2. Schematic for development of TAM RORs. In total, 256 patients were eligible for analysis. Samples were randomly split into training cohort and independent validation cohort. The training cohort was used to analyze the correlation between clinicopathological factors, gene locus polymorphism as well as the recurrence and metastasis of disease. The validation cohort was used to test the performance of the algorithm model.The variables with MeanDecreaseGini score less than 2.1 were excluded; the remaining 9 variables were used for further logistic regression analysis TAM RORs (1)=-2.74+3.54Grade+0.75LN+0.28*CYP2C19*2*+0.49PR+0.31*CYP2D6*10*+1.11ER-0.1*CYP3A5*3*-0.28Ki67-0.37Size was obtained, then TAM RORs (1) was converted into binaryzation results: TAM RORs (2)= 1/(1+e-TAM RORs(1)).

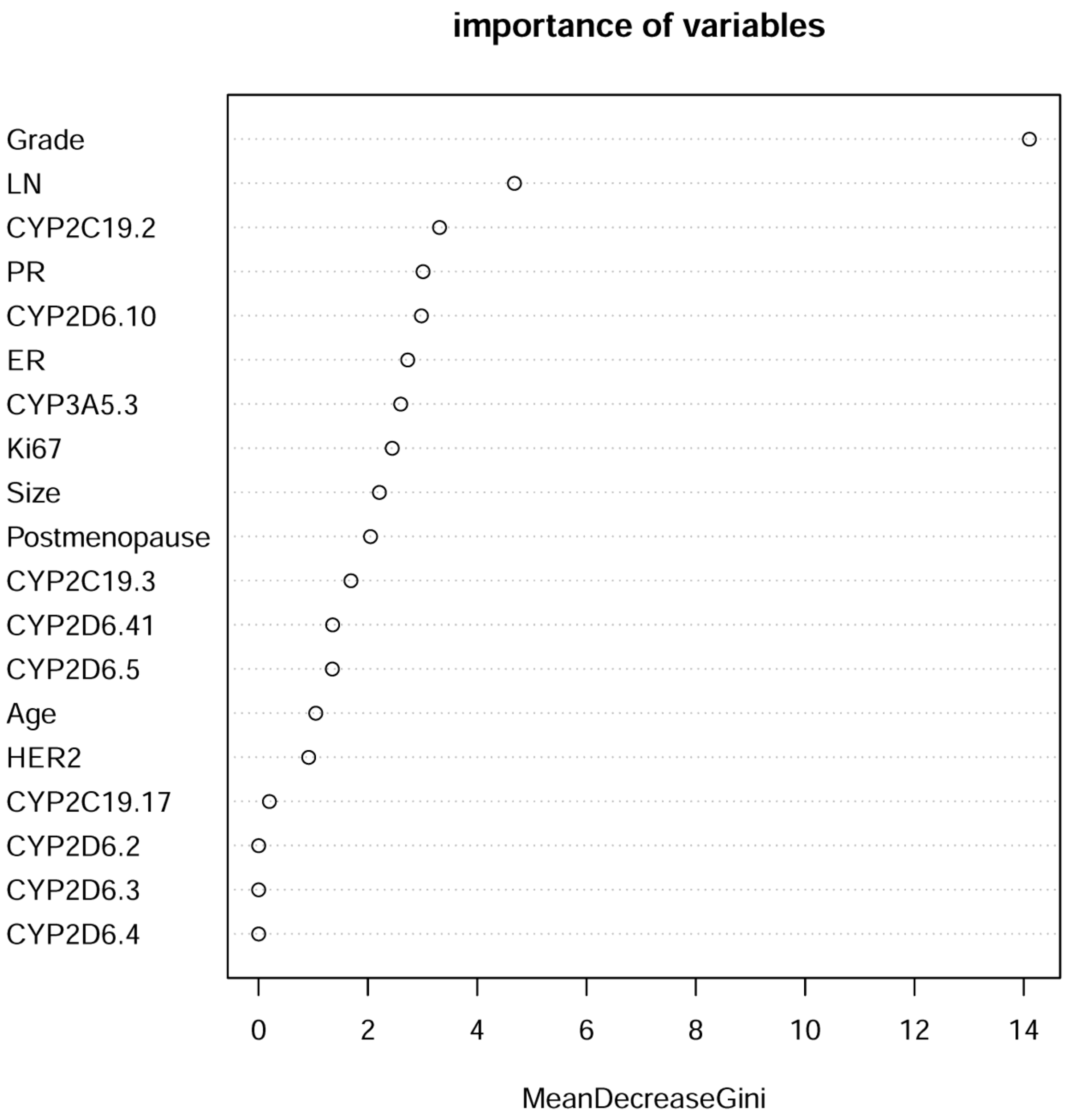

Figure S3. Variable importance of Genetic polymorphisms, clinical and pathologic features. Nineteen factors were examined for variable importance in predicting disease control rate (DCR) using Random Forest Modeling .

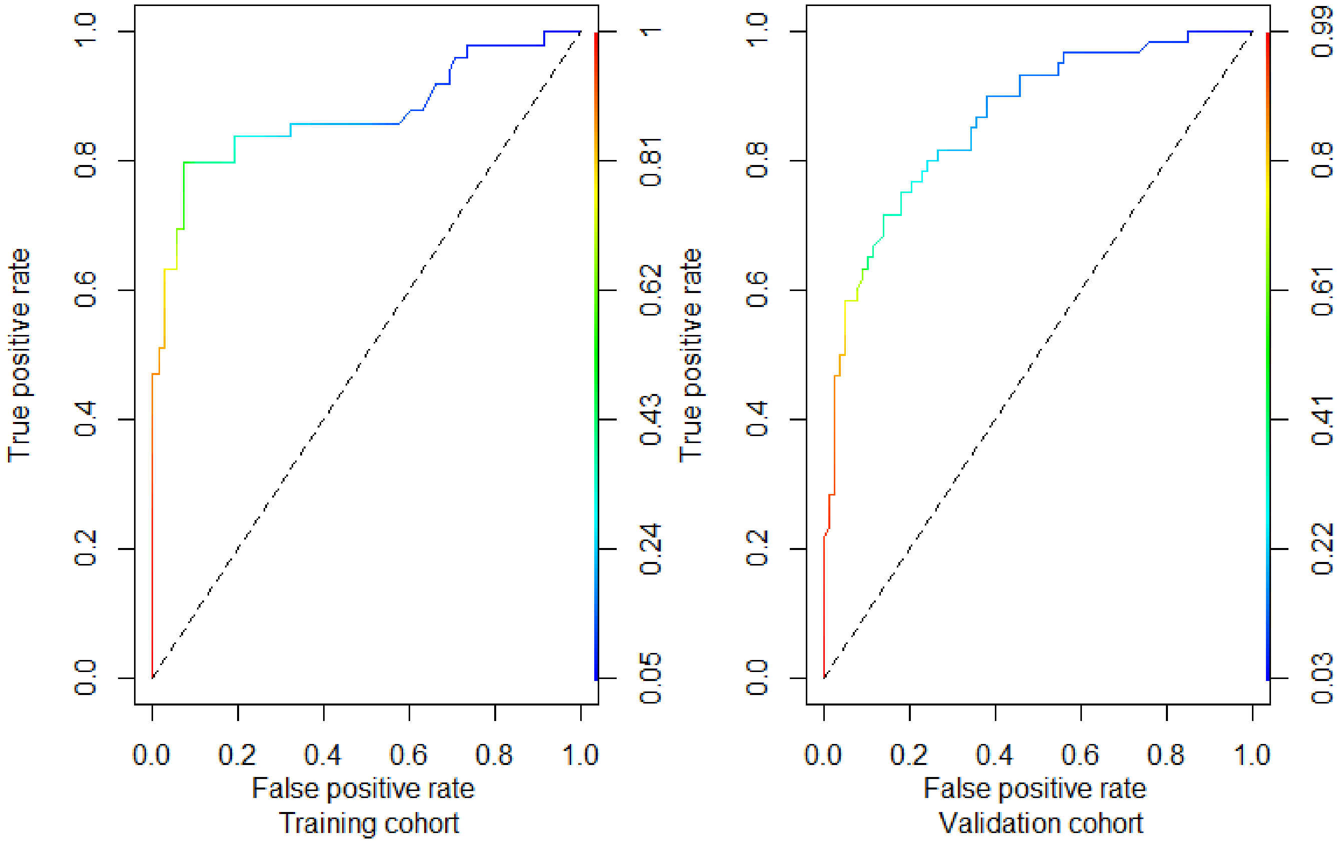

Figure S4. Receiver operating characteristic curve (ROC) of training cohort and validation cohort. The **o**rdinate means sensitivity. The abscissa means the difference between 1 and specificity.

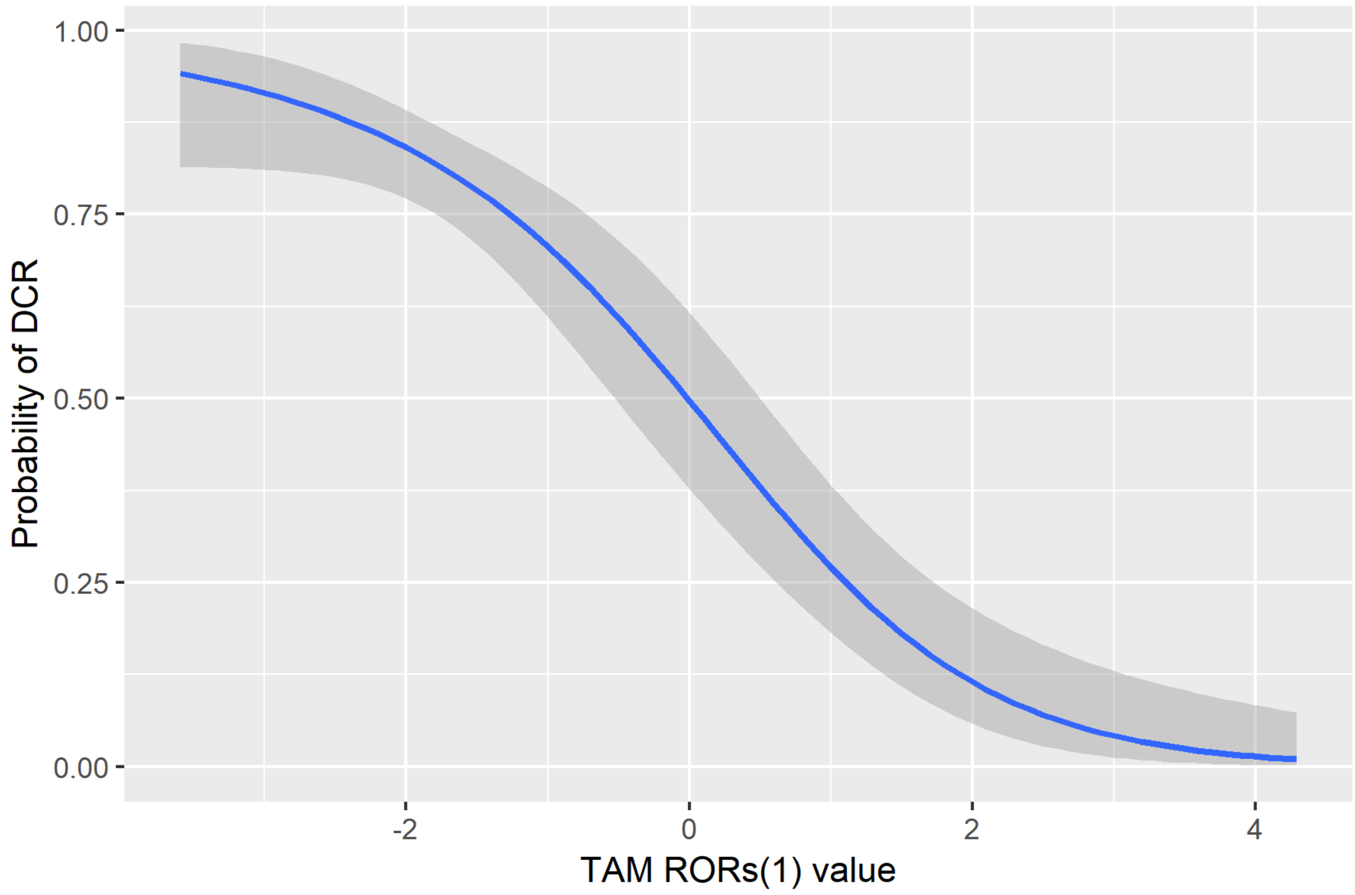

Figure S5. Receiver operating characteristic curve (ROC) of training cohort and validation cohort. Depicted are logistic regression models relating TAM RORs values to predicted disease control rate (DCR) following TAM therapy. Solid Line=fitted model; shaded region=95% confidence interval.

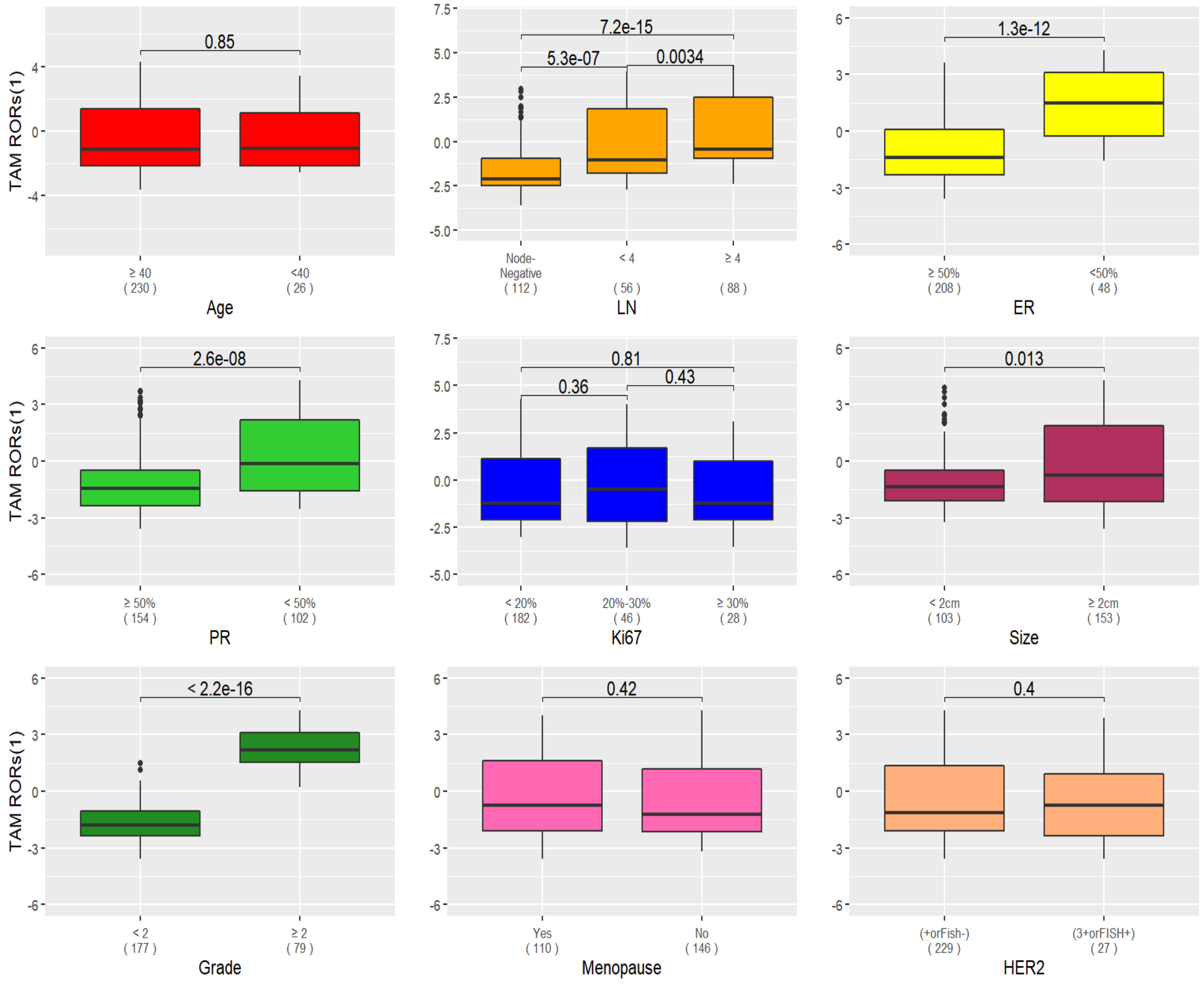

Figure S6. TAM RORs（1）associates with adverse clinical-pathologic features.

Box-whisker plots of TAM RORs values for clinical-pathologic variables (dichotomized).

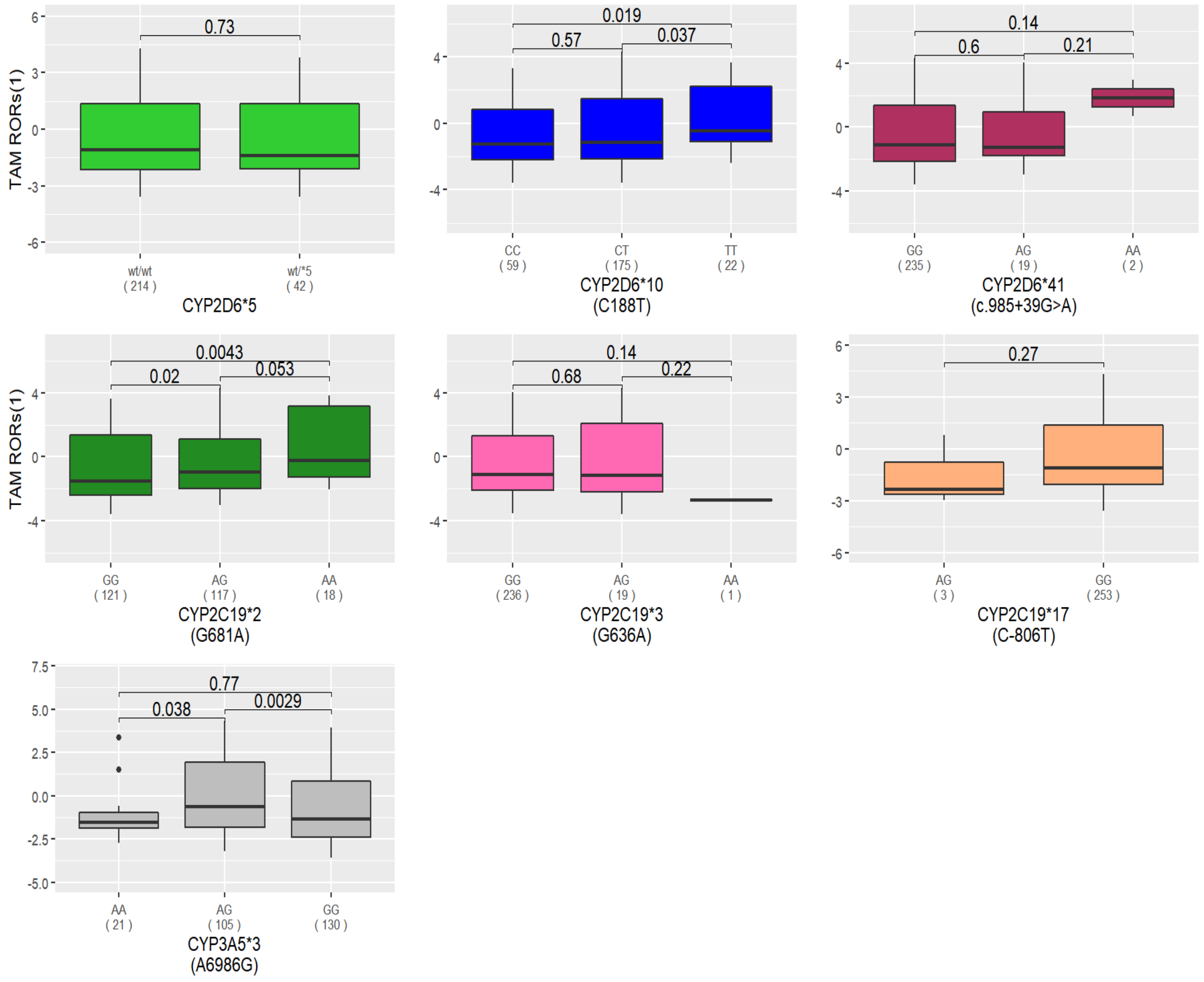

Figure S7. TAM RORs（1）associates with the genotypes.

Box-whisker plots of TAM RORs values for genotypes (dichotomized).
